# Off-resonance magnetoresistance spike in irradiated ultraclean 2D electron systems

**DOI:** 10.1186/1556-276X-8-241

**Published:** 2013-05-16

**Authors:** Jesus Iñarrea

**Affiliations:** 1 Escuela Politécnica Superior, Universidad Carlos III, Leganes, Madrid, 28911, Spain

**Keywords:** Off-resonance, Microwaves, Magnetoresistance

## Abstract

We report on the theoretical studies of a recently discovered strong radiation-induced magnetoresistance spike obtained in ultraclean two-dimensional electron systems at low temperatures. The most striking feature of this spike is that it shows up on the second harmonic of the cyclotron resonance. We apply the radiation-driven electron orbits model in the ultraclean scenario. Accordingly, we calculate the new average advanced distance by the electron in a scattering event which will define the unexpected resonance spike position. Calculated results are in good agreement with experiments.

## Background

Transport excited by radiation in a two-dimensional electron system (2DES) has been always [1-3] a central topic in basic and especially in applied research. In the last decade, it was discovered that when a high mobility 2DES in a low and perpendicular magnetic field (*B*) is irradiated, mainly with microwaves (MW), some striking effects are revealed: radiation-induced magnetoresistance (*R*_*xx*_) oscillations and zero resistance states (ZRS) [4,5]. Different theories and experiments have been proposed to explain these effects [6-18], but the physical origin is still being questioned. An interesting and challenging experimental results, recently obtained [19] and as intriguing as ZRS, consists in a strong resistance spike which shows up far off-resonance. It occurs at twice the cyclotron frequency, *w*≈2*w*_c_[19], where *w* is the radiation frequency, and *w*_c_ is the cyclotron frequency.

Remarkably, the only different feature in these experiments [19] is the use of ultraclean samples with mobility *μ* ∼ 3 × 10^7^ cm^2^ V s^-1^ and lower temperatures *T*∼0.4 K. Yet, for the previous ‘standard’ experiments and samples [4,5], mobility is lower (*μ* < 10^7^ cm^2^ V s^-1^) and *T* higher (*T* ≥ 1.0 K).

In this letter, we theoretically study this radiation-induced *R*_*xx*_ spike, applying the theory developed by the authors, the *radiation-driven electron orbits model*[6-10,20-25]. According to the theory, when a Hall bar is illuminated, the electron orbit centers perform a classical trajectory consisting in a classical forced harmonic motion along the direction of the current at the radiation frequency, *w*. This motion is damped by the interaction of electrons with the lattice ions and with the consequent emission of acoustic phonons.

We extend this model to an ultraclean sample, where the Landau levels (LL), which in principle are broadened by scattering, become very narrow. This implies an increasing number of states at the center of the LL sharing a similar energy. In between LL, the opposite happens: the density of states dramatically decreases. This will eventually affect the measured stationary current and *R*_*xx*_.

We obtain that in the ultraclean scenario, the measured current on average is the same as the one obtained in a sample with full contribution to *R*_*xx*_ but delayed as if it were irradiated with a half MW frequency (*w*/2). Accordingly, the cyclotron resonance is apparently shifted to a new *B*-position around *w* ≈ 2*w*_c_.

## Methods

The radiation-driven electron orbits model was developed to explain the *R*_*xx*_ response of an irradiated 2DEG at low magnetic field [6-10,20-25]. The corresponding time-dependent Schrödinger equation can be exactly solved. Thus, we first obtain an exact expression of the electronic wave vector for a 2DES in a perpendicular *B*, a DC electric field, and radiation:

$$\Psi_{N}(x,t)\propto \phi_{n}(x-X-x_{\text{cl}}(t),t),$$

where *ϕ*_*n*_ is the solution for the Schrödinger equation of the unforced quantum harmonic oscillator. *x*_*cl*_(*t*) is the classical solution of a forced and damped harmonic oscillator:

$$x_{\text{cl}}=\frac{eE_{o}}{m^{*}\sqrt{{\left(w_{c}^{2}-w^{2}\right)}^{2}+\gamma^{4}}}\cos\text{wt}=A\cos\text{wt},$$

where *E*_0_ is the MW electric field, and *γ* is a damping factor for the electronic interaction with the lattice ions. Then, the obtained wave function is the same as the standard harmonic oscillator, where the center is displaced by *x*_*cl*_(*t*). Next, we apply time-dependent first-order perturbation theory to calculate the elastic charged impurity scattering rate between the two *oscillating* Landau states, the initial *Ψ*_*n*_, and the final state *Ψ*_*m*_[6-10,20-24]: *W*_*n*,*m*_ = 1 / *τ*, with *τ* being the elastic charged impurity scattering time.

We find that the average effective distance advanced by the electron in every scattering jump [6-10,20-24],

*ΔX*^MW^ = *ΔX*^0^ + *A* cos*wτ*, where *ΔX*^0^, is the advanced distance in the dark [26]. Finally, the longitudinal conductivity *σ*_*xx*_ is given by,

(1) $$\sigma_{\text{xx}}\propto \int \text{dE}\frac{\Delta X^{\text{MW}}}{\tau }=\int \text{dE}\frac{\Delta X^{0}+A\cos w\tau }{\tau },$$

with *E* being the energy [26], and $\frac{\Delta X^{\text{MW}}}{\tau }$ the average electron drift velocity. To obtain *R*_*xx*_, we use the usual tensor relationships $R_{\text{xx}}=\frac{\sigma_{\text{xx}}}{\sigma_{\text{xx}}^{2}+\sigma_{\text{xy}}^{2}}≃\frac{\sigma_{\text{xx}}}{\sigma_{\text{xy}}^{2}}$.

Importantly, resistance is directly proportional to conductivity: *R*_*xx*_∝*σ*_*xx*_. Thus, finally, the dependence of the magnetoresistance with radiation is given by:

$$R_{\text{xx}}\propto A\cos w\tau .$$

## Results and discussion

**Figure 1:**
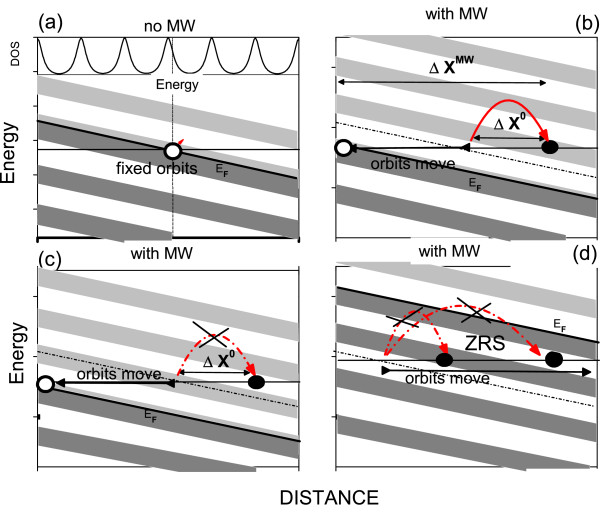
**Schematic diagrams of electronic transport for a ultraclean sample (narrow Landau levels and weak overlapping).** (**a**) In the lower part, no MW field is present. (**b**) The orbits move backwards during the jump, and the scattering ends around the central part of a LL (grey stripes); then, we have full contribution to the current. (**c**) The scattering jump ends in between LL (white stripes), giving rise to a negligible contribution to the current because the low density of final Landau states. (**d**) We depict a ZRS situation. Dotted line represents the Fermi level before the scattering jump; white and black circles represent empty and occupied orbits after the jump, respectively.

For ultraclean samples, *γ* is very small; for experimental magnetic fields [19], $\Gamma <\hbar w_{c}$. This condition will dramatically affect the average advanced distance by electron in every scattering process. In contrast with standard samples where electrons always find available empty states where to be scattered, in ultraclean samples, we can clearly find two different scenarios that are described in Figure 1.

In the four panels of energy versus distance, the grey stripes are LL tilted by the action of the DC electric field in the *x* direction. Here, LL are narrow ($\Gamma <\hbar w_{c}$) and hardly overlap each other, leaving regions with a low density of states in between (white stripes). Therefore, we can observe regularly alternating grey (many states) and white (few states) stripes equally spread out. The first scenario corresponds (see Figure 1b) to an electron being scattered to the central part of a LL. As a result, the scattering can be completed with empty states to be occupied; we obtain full contribution to the conductivity and *R*_*xx*_. In Figure 1c, we describe the second scenario where the electron scatters to a region in between LL with a very low density of states. Obviously, in this case, there is no much contribution to the average or stationary current. In Figure 1d, the scattering is not efficient because the final Landau state is occupied. Both regimes, ‘in-between LL’ and ‘center of LL’, are distributed equally and alternately along one cycle of the MW-driven electron orbit motion; then, only in one-half of the cycle, we would obtain a net contribution to the current or *R*_*xx*_.

This situation is physically equivalent to having a half amplitude harmonic motion of frequency *w*. On the other hand, it is well known that for a simple harmonic motion, it is fulfilled that averaging in one cycle, $\left|\frac{A}{2}\cos\text{wt}\right|=\left|A\cos\frac{w}{2}t\right|$. Adapting this condition to our specific case, our MW-driven (forced) harmonic motion can be perceived on average as a forced harmonic motion of whole amplitude (full scattering contribution during the whole cycle) and half frequency:

$$\left|\frac{A}{2}\cos w\tau \right|≃\left|A_{2}\cos\frac{w}{2}\tau \right|,$$

being, $A_{2}=\frac{eE_{o}}{m^{*}\sqrt{{\left(w_{c}^{2}-{\left(\frac{w}{2}\right)}^{2}\right)}^{2}+\gamma^{4}}}$ and $A=\frac{eE_{o}}{m^{*}\sqrt{{\left(w_{c}^{2}-w^{2}\right)}^{2}+\gamma^{4}}}$.The last equation is only fulfilled when *A* ≃ *A*_2_, which is a good approximation according to the experimental parameters [19], (*T* = 0.4 K, *B* ≤ 0.4 T,*w*=101 GHz and MW power *P* ∼ 0.4-1 mW). With these parameters, we obtain that the amplitudes *A* and *A*_2_ are similar and of the order of 10^-6^ to 10^7^ m. The consequence is that the *ultraclean* harmonic motion (electron orbit center displacement) behaves as if the electrons were driven by the radiation of half frequency. Therefore, applying next the theory [6-10] for the ultraclean scenario, it is straightforward to reach an expression for magnetoresistance:

$$R_{\text{xx}}\propto \frac{eE_{o}}{m^{*}\sqrt{{\left(w_{c}^{2}-{\left(\frac{w}{2}\right)}^{2}\right)}^{2}+\gamma^{4}}}\cos\frac{w}{2}\mathrm{\tau .}$$

According to it, now the resonance in *R*_*xx*_ will take place at *w* ≈ 2*w*_c_, as experimentally obtained [19]. The intensity of the *R*_*xx*_ spike will depend on the relative value of the frequency term, ($w_{c}^{2}-{\left(\frac{w}{2}\right)}^{2}$), and the damping parameter *γ* in the denominator of the latter *R*_*xx*_ expression. When *γ* leads the denominator, the spike is smeared out. Yet, in situations where *γ* is smaller than the frequency term, the resonance effect will be more visible, and the spike will show up.

The damping parameter *γ* is given, after some lengthy algebra, by [27]:

$$\begin{array}{ll} \gamma =\frac{1}{\tau_{\text{ac}}} & \propto T\times \frac{2\text{eB}}{h}\overset{\infty }{\underset{m=0}{\sum }}\frac{1}{\Pi }\left[\frac{\Gamma }{{\left(E_{n}-\hbar w_{\text{ac}}-E_{m}\right)}^{2}+\Gamma^{2}}\right]\\ & \propto T\times \left(\frac{1-e^{\frac{-\Pi \Gamma }{\hbar w_{c}}}}{1+e^{\frac{-\Pi \Gamma }{\hbar w_{c}}}}\right), \end{array}$$

where *w*_ac_ is the frequency of the acoustic phonons for the experimental parameters [19].For ultraclean samples *γ* is small [19], and according to the last expression, this makes also the term inside the brackets and *γ* smaller [28-30]. In other words, it makes the damping by acoustic phonon emission and the release of the absorbed energy to the lattice increasingly difficult. Therefore, we have a *bottleneck effect* for the emission of acoustic phonons. Now, it is possible to reach a situation where ${\left(w_{c}^{2}-{\left(\frac{w}{2}\right)}^{2}\right)}^{2}≳\gamma^{4}$, making a resonance effect visible and, therefore, giving rise to a strong resonance peak at *w* ≈ 2*w*_c_.

**Figure 2:**
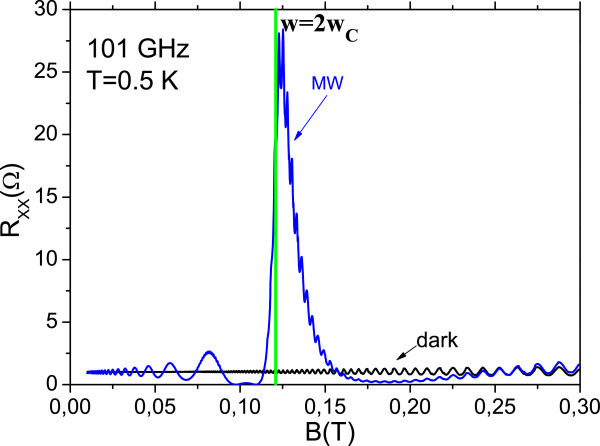
**Calculated irradiated magnetoresistance versus static magnetic field for a radiation frequency of *f *= 101 GHz.** The dark curve is also presented. For a temperature of 0.4 K, we observe an intense spike at *w* ≈ 2*w*_c_.

In Figure 2, we present a calculated irradiated *R*_*xx*_ vs. static magnetic field for a radiation frequency of *f* = 101 GHz. The curve or a dark situation is also presented. For a temperature *T* = 0.4 K, we obtain a strong spike at *w* ≈ 2*w*_c_ as in the experiments by [19].

Finally, we obtain the usual radiation-induced *R*_*xx*_ oscillations and ZRS as in standard samples.

## Conclusions

In this letter, we have presented a theoretical approach to the striking result of the magnetoresistance spike in the second harmonic of the cyclotron frequency. According to our model, the strong change in the density of Landau states in ultraclean samples affects dramatically the electron impurity scattering and eventually the conductivity. The final result is that the scattered electrons perceive radiation as of half frequency. The calculated results are in good agreement with experiments.

## Competing interests

The author declares that he has no competing interests.

## Authors’ information

JI is an associate professor at the University Carlos III of Madrid. He is currently studying the effect of radiation on two-dimensional electron systems.
